# White-Matter Pathways for Statistical Learning of Temporal Structures

**DOI:** 10.1523/ENEURO.0382-17.2018

**Published:** 2018-07-17

**Authors:** Vasilis M. Karlaftis, Rui Wang, Yuan Shen, Peter Tino, Guy Williams, Andrew E. Welchman, Zoe Kourtzi

**Affiliations:** 1 Department of Psychology, University of Cambridge, Cambridge, United Kingdom CB2 3EB; 2 Key Laboratory of Mental Health, Institute of Psychology, Chinese Academy of Sciences, Beijing, China 100101; 3 Department of Computing and Technology, Nottingham Trent University, Nottingham, NG11 8NS, United Kingdom; 4 School of Computer Science, University of Birmingham, Birmingham B15 2TT, United Kingdom; 5 Wolfson Brain Imaging Centre, University of Cambridge, Cambridge, CB2 0QQ, United Kingdom

**Keywords:** brain imaging, brain plasticity, diffusion tensor imaging, statistical learning, vision

## Abstract

Extracting the statistics of event streams in natural environments is critical for interpreting current events and predicting future ones. The brain is known to rapidly find structure and meaning in unfamiliar streams of sensory experience, often by mere exposure to the environment (i.e., without explicit feedback). Yet, we know little about the brain pathways that support this type of statistical learning. Here, we test whether changes in white-matter (WM) connectivity due to training relate to our ability to extract temporal regularities. By combining behavioral training and diffusion tensor imaging (DTI), we demonstrate that humans adapt to the environment’s statistics as they change over time from simple repetition to probabilistic combinations. In particular, we show that learning relates to the decision strategy that individuals adopt when extracting temporal statistics. We next test for learning-dependent changes in WM connectivity and ask whether they relate to individual variability in decision strategy. Our DTI results provide evidence for dissociable WM pathways that relate to individual strategy: extracting the exact sequence statistics (i.e., matching) relates to connectivity changes between caudate and hippocampus, while selecting the most probable outcomes in a given context (i.e., maximizing) relates to connectivity changes between prefrontal, cingulate and basal ganglia (caudate, putamen) regions. Thus, our findings provide evidence for distinct cortico-striatal circuits that show learning-dependent changes of WM connectivity and support individual ability to learn behaviorally-relevant statistics.

## Significance Statement

Training is known to improve performance in a range of sensory-motor tasks and alter white-matter (WM) connectivity, as measured by diffusion tensor imaging (DTI). Yet, learning to extract the statistics of event streams in natural environments is thought to often occur without explicit feedback (i.e., by mere exposure to the environment). Here, we demonstrate that this type of statistical learning of temporal structures without trial-by-trial feedback relates to changes in WM connectivity in the human brain. Our findings provide evidence for distinct cortico-striatal circuits that support individual ability to learn behaviorally-relevant statistics. In particular, individuals engage dissociable structural brain networks depending on their decision strategy, suggesting alternate brain routes to learning predictive structures.

## Introduction

Interacting successfully in dynamic environments entails that we extract meaningful structure from initially incomprehensible streams of events. This ability to extract spatial and temporal regularities from the environment, often without explicit feedback, is known as statistical learning (Perruchet and Pacton, 2006; Aslin and Newport, 2012). In particular, observers report that stimuli (shapes, tones, or syllables) that co-occur spatially or follow in a temporal sequence appear familiar (Saffran et al., 1996, 1999; Chun, 2000; Fiser and Aslin, 2002; Turk-Browne et al., 2005). Typically, regularities in the natural environment are probabilistic; for instance, combinations of sounds or syllables appear at different frequencies in the context of music or language. Learning such sequences entails extracting the probabilistic statistics that govern the temporal structure of events. Previous work has highlighted the role of strategies in probabilistic learning (Shanks et al., 2002; Erev and Barron, 2005) and perceptual decision making (Eckstein et al., 2013; Acerbi et al., 2014; Murray et al., 2015). That is, observers are shown to match their choices stochastically according to the underlying input statistics or maximize their success by selecting the most probable outcomes. Despite the fundamental importance of statistical learning for making perceptual decisions, we know surprisingly little about the brain pathways that support individual ability and strategies for learning temporal regularities.

Here, we combine behavioral measurements and multi-session diffusion tensor imaging (DTI; before and after training) to investigate the structural [i.e., white matter (WM)] pathways that engage in statistical learning of temporal structures. Recent advances in DTI allow us to reliably measure brain connectivity as indexed by local water molecule diffusion (Basser and Pierpaoli, 1996; Le Bihan et al., 2001) or long-distance brain connections (Basser et al., 2000). DTI work provides accumulating evidence for learning-dependent changes in WM connectivity (Zatorre et al., 2012) due to training in a range of tasks including motor learning (Scholz et al., 2009; Taubert et al., 2010; Sampaio-Baptista et al., 2013), spatial navigation (Sagi et al., 2012; Hofstetter et al., 2013), working memory (Takeuchi et al., 2010), artificial grammar learning (Flöel et al., 2009), and language (Schlegel et al., 2012; Hofstetter et al., 2016). Here, we ask whether mere exposure to streams of information (i.e., without trial-by-trial feedback) changes WM connectivity in pathways that support our ability to extract statistical regularities. Further, we test whether these learning-dependent changes in WM connectivity relate to individual decision strategies when learning temporal structures.

**Figure 1.**
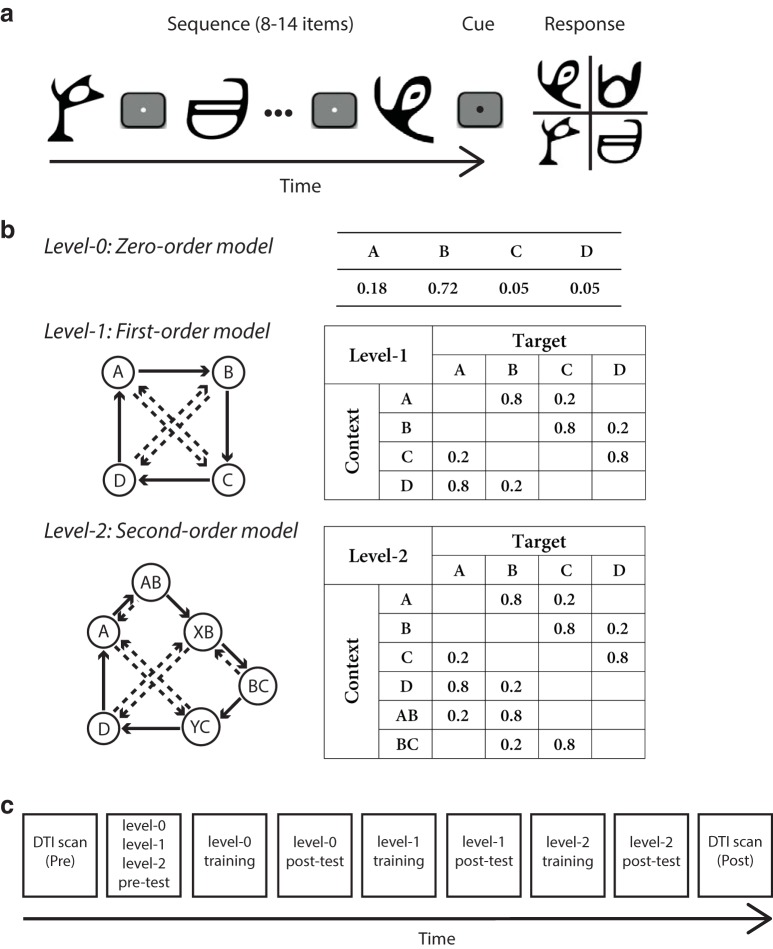
Trial and sequence design. ***A***, The trial design: 8–14 symbols were presented sequentially followed by a cue and the test display. ***B***, Sequence design: Markov models of the three context-length levels. For the zero-order model (level-0): different states (A, B, C, D) are assigned to four symbols with different probabilities. For first-order (level-1) and second-order (level-2) models, diagrams indicate states (circles) and conditional probabilities (solid arrow: high probability; dashed arrow: low probability). Transitional probabilities are shown in a four-by-four (level-1) or four-by-six (level-2) conditional probability matrix, where rows indicate the temporal context and columns the corresponding target. ***C***, Timeline of the imaging and behavioral sessions included in the study. Training involved three to five sessions for each level. DTI scans and behavioral test sessions were completed on a single day.

In particular, to investigate the brain pathways involved in learning temporal structures unencumbered by past experience, we generated temporal sequences based on Markov models of different orders (i.e., context lengths of 0, 1, or 2 previous items; Fig. 1). To simulate event structures in the natural environment that typically contain regularities at different scales, from simple repetition to probabilistic combinations, we exposed participants to sequences of unfamiliar symbols and varied the sequence structure unbeknownst to the participants by increasing the context length. We presented participants first with sequences determined by frequency statistics (i.e., occurrence probability per symbol), followed by sequences determined by context-based statistics that increased in context length (i.e., the probability of a given symbol appearing depends on the *n* preceding symbols). Participants performed a prediction task, indicating which symbol they expected to appear next in the sequence. Following previous statistical learning paradigms, participants were exposed to the sequences without trial-by-trial feedback.

Our behavioral results show that individuals adapt to the environment’s statistics, that is, they are able to extract predictive structures that change over time. Further, we show that individual learning of structures relates to decision strategy. In particular, learning context-based statistics relates to selecting the most probable outcomes in a given context (i.e., maximizing) rather than the exact sequence statistics (i.e., matching). Our DTI results demonstrate that individual strategies for learning behaviorally-relevant statistics engage distinct cortico-striatal circuits. In particular, learning-dependent changes in WM connectivity relate to individual variability in decision strategy: matching relates to connectivity changes between caudate and hippocampus, while maximizing relates to connectivity changes between prefrontal, cingulate and basal ganglia (caudate, putamen). Thus, our findings provide evidence for learning-dependent changes of WM connectivity in distinct cortico-striatal circuits that support our ability to extract behaviorally-relevant statistics in variable environments.

## Materials and Methods

### Observers

Forty-four healthy volunteers (15 female, 29 male) participated in the experiment; half participated in the training group and the rest in the no-training control group. The data from one participant per group were excluded from the study due to excessive head movement, resulting in twenty-one participants per group (training group: mean age, 21.56 years and SD, 1.84 years; no-training group: mean age, 25.53 years and SD, 2.60 years). All participants were naive to the study, had normal or corrected-to-normal vision and signed an informed consent. The training experiment was conducted in the School of Psychology, University of Birmingham and the no-training control experiment was conducted in the Department of Psychology, University of Cambridge. Both experiments were approved by the respective University Ethics Committees.

### Stimuli

Stimuli comprised four symbols chosen from Ndjuká syllabary (Fig. 1*A*). These symbols were highly discriminable from each other and were unfamiliar to the participants. Each symbol subtended 8.5^°^ of visual angle and was presented in black on a mid-gray background. Experiments were controlled using Matlab and the Psychophysics toolbox 3 (Brainard, 1997; Pelli, 1997). For the behavioral training sessions, stimuli were presented on a 21-inch CRT monitor (ViewSonic P225f 1280 × 1024 pixel, 85-Hz frame rate) at a distance of 45 cm. For the test sessions, stimuli were presented inside the MRI scanner using a projector and a mirror set-up (1280 × 1024 pixel, 60-Hz frame rate) at a viewing distance of 67.5 cm. The physical size of the stimuli was adjusted so that the angular size was constant during training and test sessions.

### Sequence design

We generated probabilistic sequences by using a temporal Markov model and varying the memory length (i.e., context length) of the sequence (Wang et al., 2017a). The model consists of a series of symbols, where the symbol at time *i* is determined probabilistically by the previous *k* symbols. We refer to the symbol presented at time *i*, *s(i)*, as the target and to the preceding *k*-tuple of symbols (*s(i-1), s(i-2), …, s(i-k)*) as the context. The value of *k* is the order or level of the sequence:

P(s(i)|s(i-1),s(i-2),…,s(1))=P(s(i)|s(i-1),s(i-2),…,s(i-k)),k<i⁡

In our study, we used three levels of memory length; for *k = 0,1,2*. The simplest *k = 0^th^* order model is a memory-less source. This generates, at each time step *i*, a symbol according to symbol probability *P(s)*, without taking into account the context (i.e., previously generated symbols). The order *k = 1* Markov model generates symbol *s(i)* at each time *i* conditional on the previously generated symbol *s(i-1)*. This introduces a memory in the sequence; i.e., the probability of a particular symbol at time *i* strongly depends on the preceding symbol *s(i-1)*. Unconditional symbol probabilities *P(s(i))* for the case *k = 0* are now replaced with conditional ones, *P(s(i)*|*s(i-1))*. Similarly, an order *k = 2* Markov model generates a symbol *s(i)* at each time *i* conditional on the two previously generated symbols *s(i-1)*, *s(i-2)*: *P(s(i)*|*s(i-1)*,*s(i-2))*.

At each time, the symbol that follows a given context is determined probabilistically, thus generating stochastic Markov sequences. The underlying Markov model can be represented through the associated context-conditional target probabilities. We used four symbols that we refer to as items A, B, C, and D. The correspondence between items and symbols was counterbalanced across participants. Note, that we designed the stochastic sources from which the sequences were generated so that the memory-conditional uncertainty remains the same across levels. In particular, for the zero-order source, only two symbols are likely to occur most of the time; the remaining two symbols have very low probability (0.05); this is introduced to ensure that there is no difference in the number of symbols across levels. Of the two dominant symbols, one is more probable (probability 0.72) than the other (probability 0.18). This structure is preserved in Markov chain of order 1 and 2, where conditional on the previous symbols, only two symbols are allowed to follow, one with higher probability (0.80) than the other (0.20). This ensures that the structure of the generated sequences across levels differs mainly in the memory length (i.e., context length) rather than the context-conditional probabilities.

In particular, for level-0, the Markov model was based on the probability of symbol occurrence: one symbol had a high probability of occurrence, one low probability, while the remaining two symbols appeared rarely (Fig. 1*B*). For example, the probabilities of occurrence for the four symbols A, B, C, and D were 0.18, 0.72, 0.05, and 0.05, respectively. Presentation of a given symbol was independent of the items that preceded it. For level-1 and level-2, the target depended on one or two immediately preceding items, respectively (Fig. 1*B*). Given a context, only one of two targets could follow; one had a high probability of being presented and the other a low probability (e.g., 80% versus 20%). For example, when Symbol A was presented, only symbols B or C were allowed to follow, and B had a higher probability of occurrence than C.

### Procedure

We tested learning of temporal structures starting with sequences determined by frequency statistics (level-0) and continuing with sequences defined by context-based statistics (level-1 and level-2). Participants were initially familiarized with the task through a brief practice session (8 min) with random sequences (i.e., all four symbols were presented with equal probability 25% in a random order). Following this, participants took part in multiple behavioral training and test sessions that were conducted on different days. In addition, they participated in two DTI imaging sessions, one before the first training session and one after the last training session. Participants were trained with structured sequences and tested with both structured and random sequences to ensure that training was specific to the trained sequences.

In particular, first, participants took part in a DTI scanning session (i.e., pre-training). Following this, participants took part in the first test session (pre-test) during which they were presented with zero-, first-, and second-order sequences and random sequences. Next, participants were trained with zero-order sequences and subsequently with first-order and variable (first and second)-order sequences in multiple behavioral sessions. For each level, participants completed a minimum of three and a maximum of five training sessions (840–1400 trials) on different days. Training at each level ended when participant performance reached PI index higher than 70% (i.e., at least 25% higher than chance) and it did not change significantly for two sessions. After completion of training per level (i.e., on the following day), participants took part in a test session during which they were presented with structured sequences determined by the statistics of the trained level and random sequences (90 trials each). A day after the last test session, participants took part in the second DTI scan (i.e., post-training). The mean time interval (±SD) between the pre-training and the post-training test sessions was 23.3 (±2.5) d. The timeline of the behavioral and imaging sessions is depicted in Figure 1*C*.

### Psychophysical training

Each training session comprised five blocks of structured sequences (56 trials per block) and lasted 1 h. To ensure that sequences in each block were representative of the Markov model order per level, we generated 10,000 Markov sequences per level comprising 672 items per sequence. We then estimated the Kullback–Leibler divergence (KL divergence) as follows:

$$KL=\sum_{target}Q(target)\log\left(\frac{Q(target)}{P(target)}\right)$$

for the level-0 model, and

$$KL=\underset{context}{\sum }Q(context)\underset{target}{\sum }Q\left(target|context\right)log\left(\frac{Q(target|context)}{P(target|context)}\right)$$

for the level-1 and level-2 models, where P() refers to probabilities or conditional probabilities derived from the presented sequence and Q() refers to those specified by the ideal Markov model. We selected fifty sequences with the lowest KL divergence (i.e., these sequences matched closely the Markov model per level). The sequences presented to the participants during the experiments were selected randomly from this sequence set.

For each trial, a sequence of 8–14 symbols appeared in the center of the screen, one at a time in a continuous stream, each for 300 ms followed by a central white fixation dot (ISI) for 500 ms (Fig. 1*A*). This variable trial length ensured that participants maintained attention during the whole trial. Each block comprised equal number of trials with the same number of items. The end of each trial was indicated by a red dot cue that was presented for 500 ms. Following this, all four symbols were shown in a 2 × 2 grid. The positions of test stimuli were randomized from trial to trial. Participants were asked to indicate which symbol they expected to appear following the preceding sequence by pressing a key corresponding to the location of the predicted symbol. Participants learned a stimulus-key mapping during the familiarization phase: key “8,” “9,” “5,” and “6” in the number pad corresponded to the four positions of the test stimuli, upper left, upper right, lower left and lower right, respectively. After the participant’s response, a white circle appeared on the selected item for 300 ms to indicate the participant’s choice, followed by a fixation dot for 150 ms (ITI) before the start of the next trial. If no response was made within 2 s, a null response was recorded and the next trial started. Participants were given feedback [i.e., score in the form of performance index (PI); see below, Behavioral analysis] at the end of each block, rather than per-trial error feedback, which motivated them to continue with training.

### Test sessions

The pre-training test session (pre) included nine runs (i.e., three runs per level), the order of which was randomized across participants. Test sessions after training per level included nine runs of structured sequences determined by the same statistics as the corresponding trained level and random sequences. Each run comprised five blocks of structured and five blocks of random sequences presented in a random counterbalanced order (two trials per block a total of 10 structured and 10 random trials per run), with an additional two 16 s fixation blocks, one at the beginning and one at the end of each run. Each trial comprised a sequence of 10 stimuli, which were presented for 250 ms each, separated by a blank interval during which a white fixation dot was presented for 250 ms. Following the sequence, a response cue (central red dot) appeared on the screen for 4 s before the test display (comprising four test stimuli) appeared for 1.5 s. Participants were asked to indicate which symbol they expected to appear following the preceding sequence by pressing a key corresponding to the location of the predicted symbol. A white fixation was then presented for 5.5 s before the start of the next trial. In contrast to the training sessions, no feedback was given during test. The test sessions took place in the MRI scanner during the acquisition of fMRI data.

### DTI data acquisition

Scanning for the training experiment was conducted using a 3T Philips Achieva MRI scanner with a 32-channel head coil. T1-weighted anatomic data (175 slices; 1 × 1 × 1 mm^3^ resolution) were collected during the first scanning session and DTI data were collected in both scanning sessions (i.e., before the first and after the last training session). The DTI acquisition consisted of 60 isotropically-distributed diffusion weighted directions (b = 1500 smm^−2^; TR = 9.5 s; TE = 78 ms; 75 slices; 2 × 2 × 2 mm^3^ resolution; SENSE) plus a single volume without diffusion weighting (b = 0 smm^−2^, denoted as b0). The DTI sequence was repeated twice during each session, once following the anterior-to-posterior phase-encoding direction and once the posterior-to-anterior direction. This acquisition scheme was implemented to allow correction of susceptibility-induced geometric distortions (Andersson et al., 2003).

Scanning for the no-training control experiment was conducted using a 3T Siemens Trio MRI scanner with a 32-channel head coil. T1-weighted anatomic data (175 slices; 1 × 1 × 1 mm^3^ resolution) were collected during the first scanning session and DTI data in both scanning sessions (26.1 ± 5.2 d apart). The DTI acquisition parameters were matched as closely as possible to the training group: 60 isotropically-distributed diffusion weighted directions (b = 1500 smm^−2^; TR = 8.9 s; TE = 91 ms; 72 slices; 2 × 2 × 2 mm^3^ resolution; GRAPPA) plus a single volume without diffusion weighting (b = 0 smm^−2^). The DTI sequence was repeated twice during each session, once following the anterior-to-posterior phase-encoding direction and once the Posterior-to-Anterior direction. Each scanning session was followed by a behavioral test in the lab the following day.

### Behavioral analysis

#### Performance index (PI)

We assessed participant responses in a probabilistic manner. We computed a PI per context that quantifies the minimum overlap (min, minimum) between the distribution of participant responses and the distribution of presented targets estimated across 56 trials per block by:

$$PI(context)=\underset{s}{\sum }min(P_{resp}(s_{t}|context_{t}),P_{pres}(s_{t}|context_{t}))$$

where *t* is the trial index and the target *s* is from the symbol set A, B, C, and D.

The overall PI is then computed as the average of the performance indices across contexts, PI(context), weighted by the corresponding context probabilities:

$$PI=\underset{context}{\sum }PI(context)\cdot P(context)$$

To compare across different levels, we defined a normalized PI measure that quantifies relative participant performance above random guessing. We computed a random guess baseline, i.e., performance index PI_rand_ that reflects participant responses to targets with (1) equal probability of 25% for each target per trial for level-0 (PI_rand_ = 0.53); (2) equal probability for each target for a given context for level-1 (PI_rand_ = 0.45) and level-2 (PI_rand_ = 0.44). To correct for differences in random-guess baselines across levels, we subtracted the random guess baseline from the performance index (PI_normalized_ = PI − PI_rand_).

#### Strategy choice and strategy index

To quantify each participant’s strategy, we compared individual participant response distributions (response-based model) to two baseline models: (1) a probability matching model, where probabilistic distributions are derived from the Markov models that generated the presented sequences (model matching); and (2) a probability maximization model, where only the most likely outcomes are allowed for each context (model maximization). We used KL divergence to compare the response distribution to matching versus maximization. KL is defined as follows:

$$KL=\sum_{target}M\left(target\right)log\left(\frac{M(target)}{R(target)}\right)$$

for the level-0 model, and

$$KL=\underset{context}{\sum }M(context)\underset{target}{\sum }M\left(target|context\right)log\left(\frac{M(target|context)}{R(target|context)}\right)$$

for the level-1 and level-2 models, where R () and M () denote the probability distribution or conditional probability distribution derived from the human responses and the models (i.e., probability matching or maximization) respectively, across all the conditions.

We quantified the difference between the KL divergence from the response-based model to model matching and the KL divergence from the response-based model to model maximization. We refer to this quantity as strategy choice indicated by ΔKL (model maximization, model matching). We then derived an individual strategy index by calculating the integral of each participant’s strategy curve across trials and subtracting it from the integral of the exact matching curve across trials, as defined by model matching. We defined the integral curve difference (ICD) between individual strategy and exact matching as the individual strategy index (where 0 = matching and values higher than 0 indicate deviation from matching toward maximization).

### DTI analysis

#### Whole-brain probabilistic tractography

We used the Automated Anatomic Labeling (AAL) atlas (Tzourio-Mazoyer et al., 2002) to define three anatomic regions (vmPFC: medial orbitofrontal in AAL, putamen and caudate) in MNI space as seed regions. We then tested WM connectivity seeded from these regions bilaterally using FSL 5.0.8 to perform the following preprocessing steps: (1) artifact correction, (2) modeling of diffusion parameters with crossing fibers, (3) simulation of whole-brain probabilistic tractography, and (4) transformation of individual maps to standard space for group analysis (i.e., alignment to MNI).

We first corrected the data for susceptibility distortions, eddy currents, and motion artifacts (Andersson and Sotiropoulos, 2016) and rotated the gradient directions (bvecs) to correct for the estimated motion rotation (Leemans and Jones, 2009; Jones and Cercignani, 2010; Ersoz et al., 2014). We generated a distribution model in each voxel using *FSL BedpostX* (Behrens et al., 2003) with default parameters.

We aligned each seed region to each participant’s native space, as probabilistic tracking is conducted in the native diffusion space. We followed a four-step registration procedure: (1) aligned the non-weighted diffusion volume (b0) of each session to their midspace and create a midspace-template (rigid-body; Smith et al., 2001; Thomas and Baker, 2013), (2) aligned the midspace-template to the anatomic (T1) scan (affine), (3) aligned the T1 to the MNI template of FSL (non-linear), and (4) inverted and combined all the transformation matrices of the previous steps to obtain the MNI-to-native registration. To extract the seed regions, the final transformation matrix was applied to the AAL atlas (nearest-neighbor interpolation). The results of each step were visually inspected to ensure that the alignment was successful.

We simulated tracts (i.e., probabilistic streamlines) starting from each seed region and extending to any other area of the brain using the probabilistic tracking algorithm (*ProbtrackX*; Behrens et al., 2007). To test the connectivity from each seed area to the whole brain, we used a mid-sagittal exclusion mask to prevent tracts from crossing hemisphere (no termination or waypoint mask were used; Behrens et al., 2007). The parameters we used in *ProbtrackX* are: 5000 samples per voxel, 2000 steps per sample until conversion, 0.5-mm step length, 0.2 curvature threshold, 0.01 volume fraction threshold and loopcheck enabled to prevent tracts from forming loops.

The main output of *ProbtrackX* is a visitation map in the native space which shows the number of tracts passing through each voxel (streamline count). To control for differences in volume across seeds and participants, we estimated connection probability between each brain voxel and a seed region by dividing the streamline count by the total number of tracts started from the seed region (Johansen-Berg and Rushworth, 2009), resulting in a normalized visitation map per participant.

#### Regression analysis of WM connectivity with strategy

To perform statistical tests on the probabilistic tractography maps across participants, we aligned each participant’s normalized visitation map to MNI using trilinear interpolation. For further analysis we applied a threshold of 0.1% connection probability on this map to remove the less probable pathways and reduce the number of voxels to be tested (Schulz et al., 2015). We then binarized the connection probability map per participant and averaged the maps across participants to generate a map of voxels with connection probability higher than 0.1% in at least 50% of the participants (Cohen et al., 2008; de Wit et al., 2012; van den Brink et al., 2014) and further reduce the number of voxels considered for statistical analysis.

We used this thresholded map as a mask for the individual participant connection probability maps for each of the two test sessions (pre- and post-training). We then subtracted the pre-training connection probability map from the post-training one, resulting in a connection probability change map for each participant. To test whether connectivity in this map relates to individual behavior (i.e., strategy), we conducted nonparametric voxel-wise statistical testing using a permutation-based statistical tool, *FSL Randomise* (Winkler et al., 2014). We tested a GLM model with strategy index for frequency statistics (level-0) and strategy index for context-based statistics (mean index for level-1 and level-2) as regressors. Note that modeling the behavioral data showed that the strategy index was highly correlated between level-1 and level-2 (*r* = 0.72, *p* < 0.001^a^), while no significant correlations were observed with level-0 (level-0 versus level-1: *r* = -0.21, *p* = 0.35; level-0 versus level-2: *r* = -0.15, *p* = 0.52). To avoid including collinear predictors in the regression model (Farrar and Glauber, 1967; Hill and Adkins, 2011), we averaged the strategy index across level-1 and level-2, generating a single predictor for learning context-based statistics. This allowed us to estimate robustly the effect of each predictor (strategy for learning frequency statistics or context-based statistics) independently. The *Randomise* algorithm permutes all participants’ samples 10,000 times to generate a null-distribution based on the data; it then compares the observed data to the generated null-distribution. To determine significance we used the threshold-free cluster enhancement (TFCE) method that takes into account the spatial extent of voxel clusters (Smith and Nichols, 2009). We accepted voxels that passed multiple comparisons using Family-wise Error Rate (FWER) correction at *a* = 0.05. This analysis results in voxel clusters that are significantly correlated with each regressor (i.e., strategy for frequency or context-based statistics). Further, we present correlation plots showing connection probability change values extracted from the peak voxel of each significant cluster with strategy index of individual participants to demonstrate that our results were not driven by outliers (see Results). Note that these plots are only descriptive; no additional statistics were conducted on these data to avoid circularity.

### Statistical analysis

Statistical analyses of the behavioral and DTI data are summarized in Table 1 (superscript letters in the statistical results indicate the reported tests). In particular, voxel-wise DTI connectivity tests were performed in FSL using a permutation-based statistical tool, *FSL Randomise* (Winkler et al., 2014). We conducted repeated measures ANOVA and power calculations in IBM SPSS 25. For comparison between groups (training versus no-training control) we also conducted Bayesian statistics (repeated measures Bayesian ANOVA, Bayesian *t* test) in JASP (JASP Team 2018, JASP version 0.8.6). The Bayes factor (BF_10_) quantifies the strength of evidence in favor of the data supporting the alternative rather than null hypothesis: BF_10_ < 1 provides evidence favoring the null hypothesis (with BF_10_ between 1/10 and 1/3 providing substantial evidence for the null hypothesis; Kass and Raftery, 1995; Wagenmakers et al., 2011), while BF_10_ > 1 provides evidence favoring the alternative hypothesis.

**Table 1.** Summary of statistical analyses

	Data structure	Type of test	Power
a.	Normal distribution	Pearson correlation	99%
b.	Normal distribution	Three-way repeated measures ANOVA	Group: 17% (n.s.)
c.	Normal distribution	Two-way repeated measures ANOVA	Group: 8% (n.s.)
d.	Normal distribution	Two-sample *t* test	12% (n.s.)
e.	Normal distribution	Two-way repeated measures ANOVA	Session: 100% Level: 100%
f.	Normal distribution	Three-way repeated measures ANOVA	Group: 100% Group × session: 100%
g.	Normal distribution	Two-way repeated measures ANOVA	Group: 6% (n.s.) Group × level: 20% (n.s.)
h.	Normal distribution	One-way repeated measures ANOVA	87%
i.	Normal distribution	Fisher’s *z* test	Right putamen - IFG: 55% Left vmPFC - caudate: 52% Left caudate - hippocampus: 87%
j.	Normal distribution	Two-way repeated measures ANOVA	Group: 20% (n.s.) Group × pathway: 39% (n.s.)

Letters refer to reported tests in the Results.

### Comparison between groups

#### Data quality

The training and no-training control groups were tested at different 3T scanners (3T Phillips Achieva, 3T Siemens Trio) using highly similar sequences and scanning parameters. To ensure that the data quality was comparable across groups and control for interscanner variability we conducted the following analyses. First, we calculated the sum of squared errors (sse) from diffusion tensor model fit, that is, we used the *dtifit* algorithm (Behrens et al., 2003) to fit a diffusion tensor model per voxel and assessed the quality of the fit based on the residuals (sse of the model per voxel). We then used this data quality measure as a nuisance regressor in the analyses comparing connectivity between the two groups to ensure that differences between groups could not be simply explained by variability in DTI data quality (see Results, Comparing DTI-based connectivity between training and no-training groups). Second, we computed whole-brain WM SNR from the b0 data (i.e., DTI data without diffusion weighting) as $\sqrt{\pi /2}\frac{signal}{noise}$ (Dietrich et al., 2007), where signal is the mean value in WM, and noise is the mean value in an area outside the brain (sphere of 10-mm radius). Third, we computed whole-brain gray matter (GM) SNR from a separate T1 scan, similarly to the b0 SNR. We then compared these three data quality measures across groups using conventional and Bayesian statistics. No significant differences were observed between groups for diffusion tensor model fit for each area of interest (including all seed and target regions) as well as the whole brain and the WM (*F*_(1,40)_ = 1.05, *p* = 0.311, BF_10_ = 0.209^b^), b0 SNR (*F*_(1,40)_ = 0.25, *p* = 0.620, BF_10_ = 0.668^c^) nor T1 SNR (*t*_(40)_ = 0.76, *p* = 0.451, BF_10_ = 0.382^d^). Thus, these analyses suggest that it is unlikely that differences in DTI connectivity between groups could be due to differences in data acquisition or quality. This is supported by further analysis showing no significant differences in WM connectivity before training (pre-training scan) across groups (see Results, Comparing DTI-based connectivity between training and no-training groups).

The results of these analyses controlling for interscanner variability are consistent with several studies showing high reliability for DTI measurements within [coefficient of variation (CV) < 1%] and between (CV < 3%) the scanners used in our study: 3T Phillips Achieva, 3T Siemens Trio (Magnotta et al., 2012; Palacios et al., 2017). Further studies have shown high intrascanner reliability for these scanners, specifically for DTI measurements [3T Phillips Achieva (Jansen et al., 2007; Danielian et al., 2010; Wang et al., 2012; Jovicich et al., 2014; Grech-Sollars et al., 2015; Kamagata et al., 2015), 3T Siemens Trio (Fox et al., 2012; Huang et al., 2012)]. Similar reliability for DTI measurements has also been reported across field strength and TE/TR parameters. For example, Grech-Sollars et al. (2015) report reproducibility across both 1.5T and 3T scanners for DTI, and Palacios et al. (2017) report high interscanner reliability for different scanner models, as well as for small changes in the TE/TR. Further, interscanner variability becomes problematic when comparing a single measurement between two participant groups tested in different scanners. In contrast to previous studies, we collected two measurements per group on the same scanner. This design allows us to test the effect of training by comparing each individual participant data after training to a baseline measurement collected before training on the same scanner. This comparison requires compatible data quality across sessions. We took the following steps to ensure this. First, there is evidence that intrascanner reliability increases with higher number of gradient directions and the number of DTI acquisitions (Wang et al., 2012). We used a higher number of gradient directions (i.e., 60 directions) compared to the minimum of 30 directions that is typically used and two phase-encoding direction acquisitions. Second, it has been shown that the tract-specific analysis we performed in our study has higher reliability across sessions (Kamagata et al., 2015). Third, non-linear registration to a standard space (e.g., MNI space) has been shown to improve interscanner reliability (Vollmar et al., 2010); we included this step in our data preprocessing.

#### DTI connectivity

To compare WM connectivity between the two groups of participants (training, no-training control), we first performed whole-brain probabilistic tractography for the control group using the same seed regions, exclusion mask and parameters in *ProbtrackX*, as for the training group. We followed the same normalization steps to derive connection probability change maps which we then used for nonparametric voxel-wise regression with strategy for frequency and context-based statistics. We defined strategy index from the second test session, as there were no training data for the control group. We accepted voxels that passed multiple comparisons (FWER corrected, *a* = 0.05).

Second, to directly test for differences between groups, we performed a voxel-wise ANCOVA on the connection probability change maps with strategy for frequency and context-based statistics as predictors per group. This analysis results in voxel clusters (FWER corrected, *a* = 0.05) whose correlation with each regressor (i.e., strategy for frequency or context-based statistics) is significantly different between groups. To illustrate these results, we present correlation plots showing connection probability change values extracted from the peak voxel of each significant cluster with strategy index of individual participants per group.

Finally, we performed seed-to-target probabilistic tractography in cortico-striatal pathways related to strategy for learning frequency and context-based statistics. We focused on the pathways we identified based on the whole-brain regression analysis for the training group. In particular, we used the same seed regions and identified target regions using a sphere of 5-mm radius around the peak voxel of each significant cluster revealed by the previous analysis (Robinson et al., 2012). We used a mid-sagittal exclusion mask and the same parameters in *ProbtrackX* as in the whole-brain tractography and applied the same normalization procedure to derive connection probability maps. For each participant, we computed a single connection probability value per seed-target connection, that is, we averaged the connection probability value across voxels in the target area. Then for each group, we calculated the connection probability change (i.e., post- minus pre-training) and correlated this value with strategy index. We computed the correlations using the robust correlation toolbox (Pernet et al., 2013) which accounts for potential outliers and calculates a bootstrapped confidence interval for 1000 permutations. We then converted the *r* coefficients to z-scores using Fisher z-transform and tested whether the correlations were significantly different between groups (*a* = 0.05).

## Results

### Behavioral performance

To quantify the ability of the participants to perform the prediction task (i.e., predict the target following a sequence of symbols), we computed a PI that measures how closely the probability distribution of the participant responses matches the probability distribution of the presented symbols. This is preferable to a simple measure of accuracy because the probabilistic nature of the sequences means that the ‘correct’ upcoming symbol is not uniquely specified; thus, designating a particular choice as correct or incorrect is often arbitrary.

**Figure 2.**
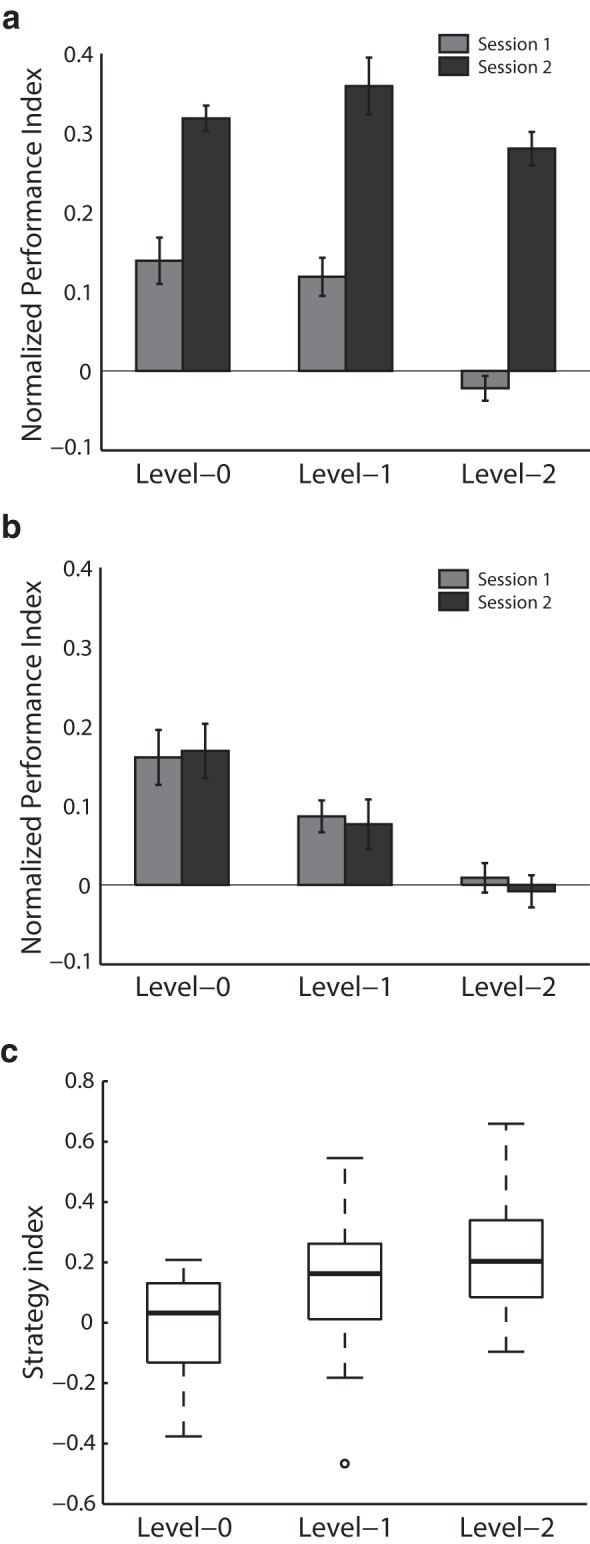
Behavioral performance. Mean normalized PI across participants per level during the first test session (gray bars) and second test session (black bars) for (***A***) the training group and (***B***) the no-training control group. Error bars indicate SEM across participants. ***C***, Strategy index boxplots for level-0, level-1, and level-2 indicate individual variability for the training group. The upper and lower error bars display the minimum and maximum data values, and the central boxes represent the interquartile range (25th to 75th percentiles). The thick line in the central boxes represents the median. Open circles denote outliers.

Comparing normalized performance (i.e., after subtracting performance based on random guessing) before and after training per level (Fig. 2*A*) showed that participants improved substantially in learning probabilistic structures. A two-way repeated measures ANOVA (Greenhouse–Geisser corrected) with session (pre, post) and level (level-0, level-1, level-2) showed a significant main effect of session (*F*_(1,20)_ = 117.9, *p* < 0.001^e^) and level (*F*_(2,40)_ = 17.9, *p* < 0.001^e^), but no significant interaction between session and level (*F*_(1.44,28.71)_ = 2.7, *p* = 0.098), suggesting enhanced performance after training and similar behavioral improvement across levels.

To test whether the behavioral improvement we observed was specific to the training and ensure that our results were not due to the participants becoming familiar with the stimuli and/or task between test sessions, we conducted a no-training control experiment. Participants in the no-training control group were tested with structured sequences in two sessions but they did not receive training in between sessions [the period between test sessions was similar for the training (23.3 ± 2.5 d) and the no-training control (26.1 ± 5.2 d) experiments]. Our behavioral results for the control group (Fig. 2*B*) showed no significant main effect of session (*F*_(1,20)_ = 0.1, *p* = 0.740) nor a significant interaction between session and level (*F*_(1.33,26.56)_ = 0.2, *p* = 0.695, Greenhouse–Geisser corrected). Comparing performance between the two groups showed a significant main effect of group (*F*_(1,40)_ = 39.0, *p* < 0.001, BF_10_ = 1083.7^f^) and a significant interaction between group and session (*F*_(1,20)_ = 73.0, *p* < 0.001, BF_10_ = 2.08·10^10f^), indicating that behavioral improvement was specific to trained sequences rather than the result of repeated exposure during the pre- and post-training sessions. Finally, comparing pre-training behavioral performance between groups showed no significant main effect of group (*F*_(1,40)_ = 0.1, *p* = 0.739, BF_10_ = 0.227^g^) nor a significant interaction between level and group (*F*_(1.43,57.36)_ = 1.0, *p* = 0.355, Greenhouse–Geisser corrected, BF_10_ = 0.317^g^), suggesting that our results are unlikely to be confounded by differences in pre-training performance.

### Decision strategies: matching versus maximization

Previous work (Shanks et al., 2002; Erev and Barron, 2005; Wozny et al., 2010; Eckstein et al., 2013; Acerbi et al., 2014; Murray et al., 2015) on perceptual decision making and probabilistic learning has proposed that individuals use two possible strategies when making a choice: matching versus maximization. In the context of our task, as the Markov models that generated stimulus sequences were stochastic, participants needed to learn the probabilities of different outcomes to succeed in the prediction task. It is possible that participants used probability maximization whereby they always select the most probable outcome in a particular context. Alternatively, participants might learn the relative probabilities of each symbol (e.g., *p*(A) = 0.18; *p*(B) = 0.72; *p*(C) = 0.05; *p*(D) = 0.05) and respond so as to reproduce this distribution, a strategy referred to as probability matching.

To quantify participants’ strategies across training, we computed a strategy index that indicates each participant’s preference (on a continuous scale, where 0 = matching and values higher than 0 indicate deviation from matching toward maximization) for responding using probability matching versus maximization (Fig. 2*C*). Box plots in Figure 2*C* indicate variability in strategy index across participants. Comparing individual strategy across levels showed a significant main effect of level (*F*_(1.44,28.79)_ = 8.0, *p* = 0.004^h^, Greenhouse–Geisser corrected) suggesting that participants’ strategy shifted closer to maximization for higher-order sequences. In particular, strategy index was higher for level-2 compared to level-0 (*t*_(19)_ = 3.6, *p* = 0.002), but not for level-2 compared to level-1 (*t*_(19)_ = 1.9, *p* = 0.066). Further, the strategy index was highly correlated between level-1 and level-2 (*r* = 0.72, *p* < 0.001; see Materials and Methods, Regression analysis of WM connectivity with strategy). We therefore calculated a mean strategy index for context-based statistics pooling data from level-1 and level-2. This mean strategy index for context-based statistics was significantly higher than the strategy index for frequency statistics (level-0; *t*_(19)_ = 2.8, *p* = 0.012). These findings suggest that participants adopted a strategy closer to maximization when learning context-based rather than frequency statistics. Note, that this relationship was not confounded by differences in performance, as there were no significant correlations between performance after training and strategy index (level-0: *r* = 0.21, *p* = 0.38; level-1: *r* = 0.06, *p* = 0.82; level-2: *r* = 0.15, *p* = 0.52).

### DTI-based connectivity analysis

**Figure 3.**
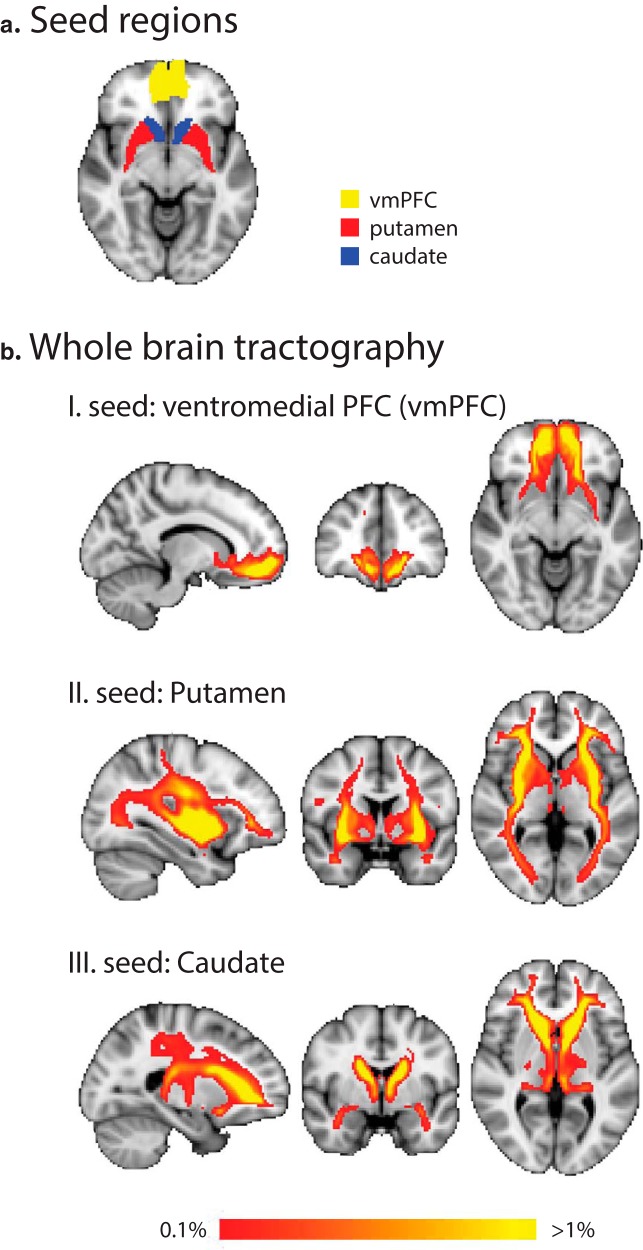
Seed regions and connection probability maps. ***A***, Seed regions for probabilistic tractography overlaid on the MNI template (*z* = -8). ***B***, Connection probability maps for each seed region (vmPFC, putamen, caudate). Maps (radiologic convention: left is right) are thresholded at 0.1% of total tracts per seed and are averaged for pre- and post-training sessions and across participants. Results are displayed in MNI for I, vmPFC (*x* = -12, *y* = 40, *z* = -8); II, putamen (*x* = -32, *y* = 2, *z* = 2); and III, caudate (*x* = -22, *y* = -2, *z* = 4) as seeds. Whole-brain tractography was computed separately for the left and right hemisphere and for pre- and post-training sessions, and the maps were combined for visualization purposes.

To investigate WM connectivity for learning temporal structures, we conducted a connection probability analysis on the DTI data collected before and after training. Previous studies have implicated the striatum and vmPFC in reward-based learning (de Wit et al., 2012; Piray et al., 2016) as well as probabilistic and statistical learning (Schendan et al., 2003; Leaver et al., 2009; Turk-Browne et al., 2009; Foerde and Shohamy, 2011). To investigate whether statistical learning changes connectivity in cortico-striatal pathways involving these regions, we defined vmPFC, putamen and caudate as seed regions (Fig. 3*A*). We used a whole-brain probabilistic tracking method to estimate connectivity distributions of WM tracts between each seed region and the rest of the brain. This method allowed us to investigate structural connectivity between distant brain regions extending beyond local WM and/or GM changes. Figure 3*B* shows average connection probability maps across participants and sessions for each seed region. This analysis shows the following cortico-striatal pathways for each seed region in accordance with previous DTI studies (Lehéricy et al., 2004; Draganski et al., 2008; de Wit et al., 2012; Jbabdi et al., 2013; Seger, 2013): (1) tracts from vmPFC project to the head of caudate and anterior-ventral putamen via anterior corona radiata; (2) tracts from putamen project to pre-SMA via corticospinal tract, to occipital lobe via inferior longitudinal fasciculus and to ventromedial and dorsolateral PFC via anterior corona radiata; (3) tracts from caudate project to temporal lobe (including hippocampus) via thalamus and to ventromedial and dorsolateral PFC via anterior corona radiata.

We then tested whether learning-dependent changes in WM connectivity relate to individual decision strategy. There is accumulating evidence for interactions between learning and decision strategy. Previous studies have shown that experience shapes the selection of decision strategies (Rieskamp and Otto, 2006; Fulvio et al., 2014). Further, faster learning of complex structures has been shown to be associated with maximizing (i.e., selecting the most probable outcomes in a given context) rather than matching the exact sequence statistics (Wang et al., 2017a). To test for learning-dependent changes in WM connectivity that relate to decision strategy, we performed a voxel-wise regression analysis of connection probability seeded from vmPFC, putamen and caudate with strategy index. We tested for significant regressions between changes in WM connectivity (before versus after training) and individual strategy for frequency and context-based statistics (Table 2). Positive correlations indicate increased connectivity after training that relates to maximization, while negative correlations indicate increased connectivity that relates to matching.

**Table 2.** DTI regression with strategy for frequency statistics and context-based statistics

				Peak voxel				
Seed	Cluster location	Hemisphere	Cluster size	*x*	*y*	*z*	*p* value	*t* value
*Frequency statistics*								
vmPFC	vmPFC, ACC, caudate (head)	L	130	-14	36	-12	0.006	5.12
vmPFC	vmPFC, ACC, caudate (head)	R	129	12	24	-8	0.012	4.93
Putamen	Thalamus, putamen, IFG	R	141	28	26	12	0.016	4.63
*Context-based statistics*								
vmPFC	ACC, caudate (head)	L	20	-14	28	-6	0.040	3.51
Caudate	Caudate (body/tail), thalamus, hippocampus, postcentral	L	214	-24	-34	6	0.022	-3.82
Caudate	Caudate, thalamus, hippocampus/lingual	L	115	-8	6	0	0.025	-4.83

Voxel-wise regression was run on connection probability change (subtracting pre- from post-training connection probability maps) with strategy index. Significance was determined using the TFCE method and corrected for multiple comparisons with FWER under random field theory for *a* = 0.05. Seed regions were selected from the AAL atlas, and the clusters were labeled using the Atlas of the Human Brain (Mai et al., 1997). The MNI coordinates, the *p* value, and the *t* value of the peak voxel are shown.

**Figure 4.**
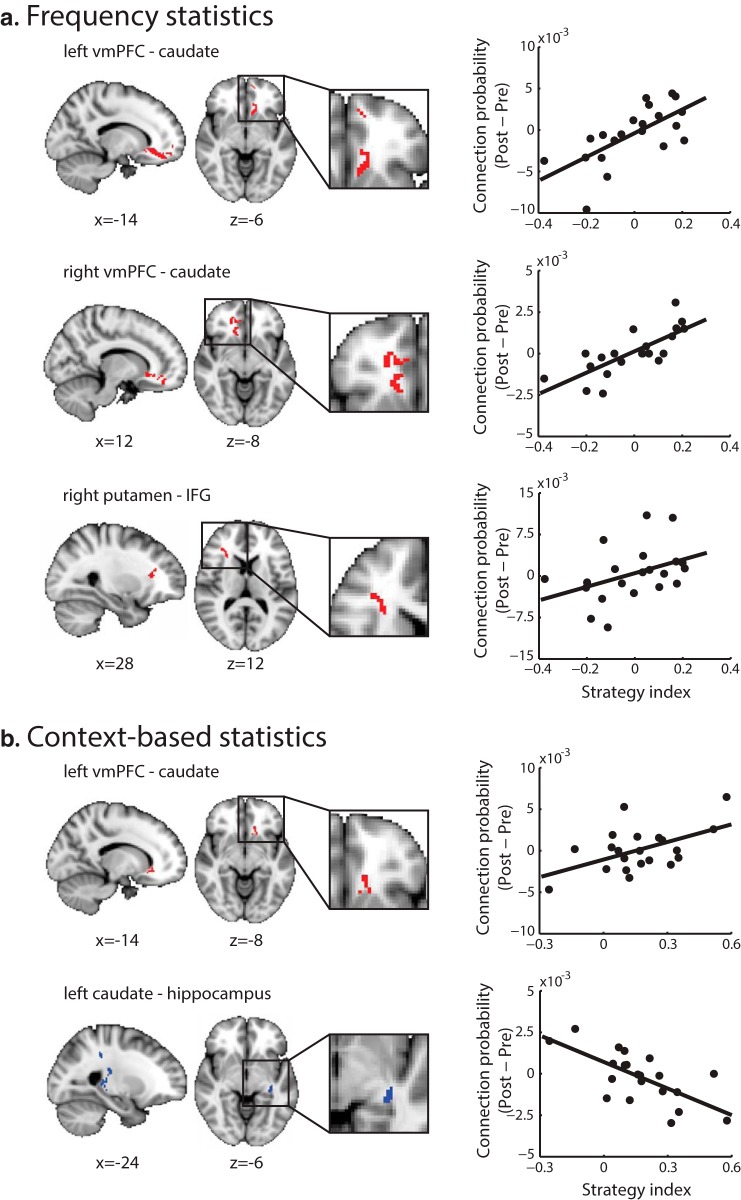
DTI regression with strategy. Clusters showing significantly positive (red clusters) or negative (blue clusters) regressions of connection probability change (post- minus pre-training) with strategy index calculated across all trials during training per level. ***A***, For learning frequency statistics, clusters comprise left vmPFC to caudate, right vmPFC to caudate and right putamen to IFG. ***B***, For learning context-based statistics, clusters comprise left vmPFC to caudate and left caudate to hippocampus. Results are displayed in radiologic convention (left is right) and are overlaid on the MNI template. An enlarged view of each significant cluster is displayed for better visibility. Scatterplots of connection probability change (post- minus pre-training) with strategy index for the peak voxel of each cluster are shown on the right panel.

Seeding from vmPFC, we found significant bilateral clusters extending from the seed to the head of caudate through anterior cingulate (ACC). These clusters showed a positive correlation between changes in connection probability with training and strategy index for learning frequency and context-based statistics (Fig. 4). For learning frequency statistics (Fig. 4*A*) this correlation was observed bilaterally, while for learning context-based statistics (Fig. 4*B*) the spatial extent of this cluster was smaller and observed only in the left hemisphere, extending from ACC to the head of caudate. These positive correlations suggest that increased connectivity after training in this pathway relates to learning by maximizing. Previous work has provided evidence for both anatomic (Lehéricy et al., 2004; Seger, 2009) and functional connectivity among these brain regions (Postuma and Dagher, 2006; Tanaka et al., 2008; Kahnt et al., 2012) that are known to be part of the motivational cortico-striatal pathway. Our findings are consistent with the role of vmPFC, ACC, and caudate in goal-directed actions (Valentin et al., 2007; Gläscher et al., 2009; Balleine and O’Doherty, 2010; de Wit et al., 2012; Levy and Glimcher, 2012) and individual strategy choice (Piray et al., 2016).

Seeding from putamen, we found a cluster that showed significant learning-dependent changes in connection probability extending from the right anterior putamen to inferior frontal gyrus (IFG) and to thalamus. This cluster showed a positive correlation between changes in connection probability with training and strategy index for learning frequency statistics (Fig. 4*A*). These brain regions are known to be part of the executive cortico-striatal pathway (Lawrence et al., 1998; Seger, 2009) and WM connectivity between these regions has been implicated in implicit sequence learning (Song et al., 2012) and artificial grammar learning (Flöel et al., 2009). In particular, IFG is implicated in attention (Simon et al., 2002) and rule switching (Cools et al., 2004), and its connectivity to anterior putamen has been reported by previous DTI studies (Lehéricy et al., 2004; Leh et al., 2007; Draganski et al., 2008).

Seeding from caudate, we found two clusters that showed significant learning-dependent changes in connection probability between caudate and hippocampus. These clusters showed a negative correlation between changes in connection probability with training and strategy index for learning context-based statistics (Fig. 4*B*). The first cluster extends from the body and tail of left caudate to thalamus (caudally) and then to hippocampus, with an additional branch to postcentral sulcus. The second cluster extends from left caudate through medial thalamus to posterior hippocampus (close to the anterior part of lingual gyrus). Both pathways are part of the visual cortico-striatal pathway as suggested by functional and structural connectivity studies (Cohen et al., 2009; Seger, 2009, 2013; Robinson et al., 2012). Our results suggest that increased connection probability between these areas after training relates to matching when learning context-based statistics. This finding is consistent with previous work implicating brain regions in the visual cortico-striatal pathway in categorization learning (Seger and Cincotta, 2005), sequence learning (Schendan et al., 2003; Albouy et al., 2008; Gheysen et al., 2011; Rose et al., 2011; Stillman et al., 2013; Rosenthal et al., 2016), and predictive associations (Turk-Browne et al., 2010; Hsieh et al., 2014; Hindy et al., 2016).

### Control analyses

We performed additional analyses to control for any possible tractography-related confounds, following previous studies (de Wit et al., 2012; van den Brink et al., 2014). First, we correlated strategy index with: (1) GM density in each seed area, (2) average fractional anisotropy (FA) change (i.e., post minus pre) in each significant cluster, (3) FA change in the peak voxel of each cluster, and (4) age. Further, we correlated the connection probability change in the peak voxel of each cluster with cerebral volume and age. None of the correlations were significant, making it unlikely that our results were confounded by individual variability in local GM or WM metrics.

Second, as tractography does not test directionality (i.e., whether the projections from area A to area B are afferent or efferent; Jbabdi and Johansen-Berg, 2011), we tested whether our results hold when seeding from the clusters that showed significant learning-dependent changes in our main analysis. In particular, we used bilateral caudate and right triangular IFG as seeds (defined based on the AAL atlas) for frequency statistics, whereas left caudate and left hippocampus as seeds for context-based statistics. Voxel-wise regression analysis of connection probability change with strategy index showed that we could recover similar clusters as in the main analysis (Table 2) at a lower statistical threshold (*a* = 0.05 uncorrected, cluster size > 20 voxels) with the exception of the right triangular IFG seed, which did not yield any significant clusters. However, seeding from the left triangular IFG, we found a significant cluster in lateral putamen and caudate extending to medial thalamus and showing a positive correlation with strategy for context-based statistics. Thus, this analysis suggests that our findings are connection-specific rather than seed-dependent, consistent with the known anatomic cortico-striatal connectivity (Alexander et al., 1986; Seger, 2009).

Third, we repeated the whole-brain tractography analysis with length correction enabled. This method weights the streamline count in each voxel with its distance from the seed; to compensate for the fact that the count decreases with the distance due to the probabilistic nature of the analysis (Tomassini et al., 2007). This weighting procedure assigns a higher weight for longer and lower weight for shorter connections, resulting in higher connection probability values compared to the previous analyses. Therefore, we applied a threshold of 4% connection probability (instead of 0.1%) to yield a comparable number of voxels for the regression analysis. We followed the same procedure as previously to correlate voxel-wise connection probability change with strategy index. We found similar connectivity clusters as in the main analysis (Table 2) when seeding from bilateral vmPFC and left caudate (FWER corrected) as well as right putamen (albeit at uncorrected *p* < 0.005) as seeds, suggesting our findings could not be significantly confounded by distance from the seed.

Finally, here we focused on learning-dependent changes in long-range WM connectivity, as measured by probabilistic tractography. Probabilistic tractography is an established methodology (Cohen et al., 2008; de Wit et al., 2012; van den Brink et al., 2014; van den Heuvel et al., 2016) that is grounded on biological mechanisms (van den Heuvel et al., 2015) and has been previously employed to investigate learning-dependent changes (Schlaug et al., 2009; Crossley et al., 2017). Yet, previous work (Scholz et al., 2009; Takeuchi et al., 2010; Taubert et al., 2010; Sagi et al., 2012; Schlegel et al., 2012; Hofstetter et al., 2013, 2016) has also reported changes in local WM due to training, as measured by FA. To test for local WM changes related to learning temporal statistics, we used TBSS analysis of FA maps (Smith et al., 2006). When performed on the whole brain, this analysis did not result in any significant clusters. We next conducted the same FA analysis within the pathways revealed by the whole-brain tractography: (1) left vmPFC and caudate, (2) right vmPFC and caudate, (3) right putamen and IFG, and (4) left caudate and hippocampus. We projected the voxels in these pathways on the FA skeleton, calculated a FA change map (i.e., post minus pre) and performed a voxel-wise regression with strategy index. Our results showed a significant cluster (FWER corrected, *a* = 0.05) in the left vmPFC that was positively correlated with strategy for context-based statistics. Although long-range connectivity is more relevant to the brain circuits involved in learning, our FA analysis suggests that it is possible to measure local WM changes due to training that are consistent with changes in long-range connectivity, as revealed by whole-brain tractography.

### Comparing DTI-based connectivity between training and no-training groups

To test whether the learning-dependent changes we observed in WM connectivity are specific to the training rather than reflecting familiarity with the stimuli and/or task due to exposure to multiple test sessions, we compared connection probability between the training group and a no-training control group. Our behavioral results showed improvement that is specific to the training rather than the result of repeated exposure to the sequences during the pre- and post-training sessions (Fig. 2*B*). To test for changes in whole-brain tractography for the no-training control group, we used the same seeds and regression analysis as for the training group. We correlated voxel-wise connection probability change with strategy after training, as there were no behavioral training data for the control group. This analysis showed no significant clusters for the control group (FWER corrected, *a* = 0.05), indicating that the WM connectivity changes with decision strategy (Fig. 4) are specific to the training and they could not be simply explained by the repeated exposure to temporal sequences during the pre- and post-training sessions.

Second, to directly compare between the training and the no-training control groups, we computed connection probability change (post- minus pre-training) per group for the cortico-striatal pathways identified by the whole-brain regression analysis (Fig. 4). We found two clusters showing significant differences after compared to before training (*a* = 0.05 uncorrected, cluster size > 100 voxels) in connection probability between groups extending: (1) from vmPFC to caudate and (2) from caudate to hippocampus. An additional but smaller cluster was observed extending from putamen to IFG (36 voxels). These clusters remained significant when we controlled for interscanner variability by including the residuals of the diffusion tensor model fit as nuisance regressor in the analysis. These results are consistent with our main findings (Fig. 4) and provide additional evidence for differences in cortico-striatal connectivity across groups independent of behavioral performance (i.e., strategy index). We next correlated the average connection probability change across voxels in these clusters with strategy index. For the training group, we observed a significant positive correlation of connection probability change between vmPFC and caudate for frequency statistics (*r* = 0.49, CI = [0.19, 0.75]), whereas a significant negative correlation of connection probability change between caudate and hippocampus for context-based statistics (*r* = -0.54, CI = [-0.77, -0.26]). However, we found no significant correlations for the same clusters and strategy for the no-training control group (vmPFC-caudate: *r* = 0.09, CI = [-0.44, 0.49]; caudate-hippocampus: *r* = -0.10, CI = [-0.44, 0.19]). These results corroborate our main findings providing evidence for training-specific changes in brain connectivity that relate to behavior.

**Figure 5.**
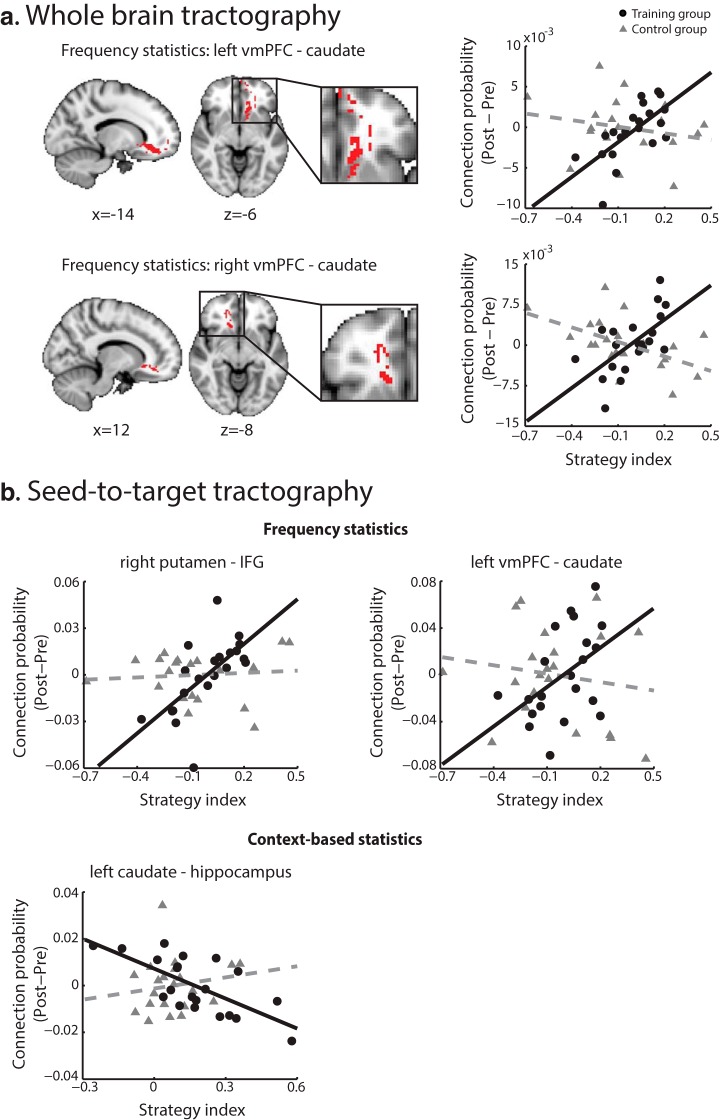
Group comparison (training versus no-training control). ***A***, Whole-brain tractography: clusters showing significantly higher correlation coefficient of connection probability change (post- minus pre-training) with strategy index for the training versus the no-training control group. Results are displayed in radiologic convention (left is right) and are overlaid on the MNI template. An enlarged view of each significant cluster is displayed for better visibility. Scatterplots of connection probability change with strategy index for the peak voxel of each cluster are shown on the right panel. ***B***, Seed-to-target tractography: correlations of connection probability change with strategy index. For learning frequency statistics, correlations were significantly different between groups for WM connectivity between right putamen and IFG (training: *r* = 0.65, CI = [0.44,0.90]; no-training control: *r* = 0.09, CI = [-0.45,0.53]) and between left vmPFC and caudate (training: *r* = 0.47, CI = [0.16,0.74]; no-training control: *r* = -0.16, CI = [-0.62,0.37]). For learning context-based statistics, correlations were significantly different between groups for WM connectivity between left caudate and hippocampus (training: *r* = -0.69, CI = [-0.86,-0.37]; no-training control: *r* = 0.16, CI = [-0.25,0.57]). Individual participant data for the training group are indicated by black circles; data for the no-training control group are indicated by gray triangles.

Third, we performed a voxel-wise ANCOVA on connection probability change maps with strategy index (frequency, context-based statistics) and group (training, no-training control). We found two significant clusters (FWER corrected, *a* = 0.05), in consistence with the main analyses of the training group data, that is, bilateral clusters extending from vmPFC (seed region) to the head of caudate through ACC. These clusters showed significantly higher correlation with strategy for frequency statistics for the training compared to the no-training control group (Table 3; Fig. 5*A*) and remained significant when we controlled for interscanner variability by including the residuals of the diffusion tensor model fit as nuisance regressor in the analysis.

**Table 3.** DTI ANCOVA between groups (training, no-training control) with strategy index

				Peak voxel				
Seed	Cluster location	Hemisphere	Cluster size	*x*	*y*	*z*	*p* value	*t* value
*Frequency statistics*								
vmPFC	vmPFC, ACC, caudate (head)	L	283	-14	36	-12	0.004	4.87
vmPFC	vmPFC, ACC, caudate (head)	R	80	18	42	-8	0.024	4.84

We conducted voxel-wise regression on connection probability change (subtracting pre- from post-training connection probability maps) with strategy index per group and tested for significant differences between groups. Significance was determined using the TFCE method and corrected for multiple comparisons with FWER under random field theory for *a* = 0.05. Seed regions were selected from the AAL atlas and the clusters were labeled using the Atlas of the Human Brain (Mai et al., 1997). The MNI coordinates, the *p* value, and the *t* value of the peak voxel are shown. Positive (negative) *t* values indicate higher (lower) correlation coefficient for the training compared to the no-training control group.

Fourth, we performed seed-to-target tractography per group using the same seeds as in the main analysis and defining the significant clusters from the main analysis as target regions (see Materials and Methods, Comparison between groups). We calculated connection probability between seed and target per test session and the difference between sessions (i.e., second minus first test session). We next correlated connection probability change (post- minus pre-training) with strategy index for each group (training, no-training control). Comparing correlations between groups (Fig. 5*B*) showed significant differences in connection probability between: (1) right putamen and IFG for learning frequency statistics (Fisher’s *z* test, *z* = 2.1, *p* = 0.037^i^); (2) left vmPFC and caudate for learning frequency statistics (Fisher’s *z* test, *z* = 2.0, *p* = 0.043^i^); (3) left caudate and hippocampus for learning context-based statistics (Fisher’s *z* test, *z* = -3.1, *p* = 0.002^i^). Comparing pre-training connection probability between groups showed no significant main effect of group (*F*_(1,40)_ = 1.3, *p* = 0.267, BF_10_ = 0.221^j^) nor a significant group × pathway interaction (*F*_(2.53,101.05)_ = 1.7, *p* = 0.185, Greenhouse–Geisser corrected, BF_10_ = 0.417^j^; DTI pathways shown in Fig. 4: left vmPFC-caudate, right vmPFC-caudate, left caudate-hippocampus, right putamen-IFG). These results show training-specific connectivity changes in key dissociable pathways that cannot be simply explained by differences in connectivity between groups (training, no-training control) before training. Taken together, these results provide evidence for training-specific changes in connection probability related to individual decision strategy.

## Discussion

Here, we sought to identify the WM pathways involved in statistical learning of temporal structures. Our behavioral results demonstrate that individuals differ in their strategy when learning to extract predictive statistics. Our DTI results demonstrate that these strategies engage distinct cortico-striatal circuits for learning behaviorally-relevant statistics. Our findings advance our understanding of the brain pathways involved in statistical learning in three main respects.

First, we provide evidence that training without trial-by-trial feedback results in changes in WM connectivity that relate to behavioral improvement in a statistical learning task. Human and animal studies have shown that DTI measurements can capture short-term (Sagi et al., 2012) and long-term (Scholz et al., 2009) WM plasticity. However, most of this work has focused on reward-based learning that involves training with trial-by-trial feedback, rather than statistical learning that occurs by mere exposure to the environment. For example, WM changes have been shown to predict behavioral performance in motor learning (Scholz et al., 2009; Taubert et al., 2010; Sampaio-Baptista et al., 2013) and reward-based learning (Cohen et al., 2008, 2009; de Wit et al., 2012). Our results are consistent with studies showing WM connectivity changes related to implicit sequence learning (Bennett et al., 2011; Song et al., 2012) in the context of a serial reaction task. However, our prediction task extends beyond sensory-motor learning or previous work using implicit measures of anticipation (i.e., RT reduction or familiarity judgments). Our paradigm allows us to directly test whether exposure to temporal sequences facilitates the observers’ ability to explicitly predict the identity of the next stimulus in a sequence. Importantly, modeling the participants’ predictions allows us to characterize individual decision strategies (matching versus maximization) when learning to extract behaviorally relevant statistics.

Second, we demonstrate that individual decision strategies engage dissociable cortico-striatal pathways (Alexander et al., 1986; Lawrence et al., 1998) that show learning-dependent changes in WM connectivity. In particular, we show that matching (i.e., extracting exact sequence statistics) relates to WM connectivity changes between the caudate, hippocampus and thalamus; these areas are known to be involved in the visual cortico-striatal pathway (Seger, 2009, 2013). In contrast, maximizing relates to WM connectivity changes between prefrontal (vmPFC), cingulate and basal ganglia (caudate) regions that are thought to be involved in the motivational cortico-striatal pathway as well as prefrontal (dorsolateral PFC: IFG) and basal ganglia (anterior putamen) regions that are thought to be involved in the executive cortico-striatal pathway. These findings are consistent with previous work showing that WM integrity or structural connectivity relate to individual variability in performance in the context of decision-making tasks. For example, connectivity in dissociable brain circuits involving hippocampal and striatal regions predicts performance in reversal learning (Cohen et al., 2008) and novelty-seeking versus reward dependence (Cohen et al., 2009). Further, connectivity in dissociable cortico-striatal circuits involved in habitual versus goal-directed learning (de Wit et al., 2012) is shown to predict individual strategy choice (Piray et al., 2016). Here, we demonstrate that learning-dependent changes of structural connectivity in these pathways relate to individual variability in decision strategy. Interestingly, there is accumulating evidence for interactions between learning and individual decision strategy (Rieskamp and Otto, 2006; Fulvio et al., 2014). Learning rate and decision strategy have been shown to be correlated, that is, faster learning of complex structures is associated with maximizing (i.e., selecting the most probable outcomes in a given context) rather than matching the exact sequence statistics (Wang et al., 2017a). Considering individual decision strategy provides further insights into individual variability in learning: we show that individuals engage dissociable structural brain networks to solve the same task depending on their decision strategy (matching versus maximization), suggesting alternate brain routes to learning predictive structures.

Recent fMRI work on learning temporal structures provides complementary evidence that functional changes in brain regions involved in these cortico-striatal pathways relate to individual decision strategies (Wang et al., 2017b). Although fMRI reveals learning-dependent changes in the processing within specific brain regions, it does not test for structural connectivity between these regions. In contrast, DTI before versus after training allows us to test for changes in the structural connectivity between nodes within a brain network, extending beyond fMRI changes in local network nodes. Our DTI findings are consistent with previous functional imaging studies showing that brain regions in the visual cortico-striatal pathway are involved in implicit sequence learning (Schendan et al., 2003; Albouy et al., 2008; Gheysen et al., 2011; Rose et al., 2011; Stillman et al., 2013; Rosenthal et al., 2016) and predictive associations (Turk-Browne et al., 2010; Hsieh et al., 2014; Hindy et al., 2016). In contrast, brain regions in the motivational cortico-striatal pathway (i.e., prefrontal and cingulate cortex) are thought to be involved in decision making, monitoring performance and switching between associations and strategies (Heekeren et al., 2008; Rushworth and Behrens, 2008) as well as predictive coding (Monchi et al., 2001; Bar, 2009). Previous work on humans and animals provides evidence for the role of caudate in switching between strategies (Monchi et al., 2001; Cools et al., 2004; Seger and Cincotta, 2006) and learning after a rule reversal (Cools et al., 2002; Pasupathy and Miller, 2005). Further, putamen, known to be involved in skilled and habitual performance (Daw et al., 2005; Balleine and O’Doherty, 2010), may facilitate learning by maximizing.

Third, our findings suggest that learning temporal structures implicates cortico-striatal pathways that are common for learning frequency and context-based statistics. Our findings show that following training connectivity in the motivational (vmPFC, ACC, caudate) and executive (IFG, putamen) cortico-striatal pathways increases for individuals who select the most probable outcome in a context. This is consistent with the role of the motivational pathway in goal-directed and model-based learning, while the role of putamen in habitual and model-free learning (Balleine and O’Doherty, 2010; de Wit et al., 2012; Piray et al., 2016). Thus, it is possible that individuals recruit goal-directed circuits to acquire temporal structures (from simple repetitive patterns to probabilistic contingencies), while habitual learning mechanisms when selecting the most probable outcome in a given context. In addition to these common pathways, learning context-based statistics involves connectivity changes between caudate and hippocampus that relate to matching. That is, extracting the exact context-target contingencies engages a pathway that is known to be involved in probabilistic learning and novelty seeking (Cohen et al., 2009; Stillman et al., 2013). As our paradigm tested learning of structures that increased in context-length over time, it does not allow us to dissociate learning time course from task demands. It would be interesting in the future to investigate the time course with which these pathways are involved in the learning of frequency and context-based statistics.

Finally, we consider our results in light of recent studies that provide controversial evidence for the efficacy of cognitive training. Several studies have shown that cognitive training improves performance on the trained task (e.g., working memory; Klingberg, 2010; Morrison and Chein, 2011); however, whether training generalizes to other tasks and has longer-term effects on cognitive performance remains debated (Owen et al., 2010; Bavelier et al., 2012). Further, several DTI studies have shown structural plasticity following cognitive training in a range of tasks: from working memory to reasoning and language learning (Mackey et al., 2012; Schlegel et al., 2012; Román et al., 2017). Yet, it remains unknown whether these structural changes due to cognitive training are long-lasting or have an effect on real-life abilities. Our study aimed to investigate the pathways involved in statistical learning of temporal structures rather than develop a cognitive training program. To this end, we trained and tested the same participants in multiple sessions. That is, we tested participants both before and after training and ensured that the training-dependent differences we observed in behavior and brain connectivity were not due to differences across participants before training. Importantly, differences in brain connectivity due to training were related to changes in behavioral performance, suggesting training-specific effects rather than brain changes related to general factors (e.g., task familiarity, motivation, task engagement). Additional evidence for training specificity came from training the same individuals with the same stimuli and task but with sequences that differed in their structure. In particular, we showed that learning frequency statistics versus context-based statistics resulted in differences in behavior (i.e., strategy) and learning-dependent changes in WM connectivity. Further, our no-training control experiment provides complimentary evidence for test-retest reliability and ensures that our results were not due to the participants simply becoming familiar with the stimuli and/or task between the two scanning sessions. We have tightly controlled for interscanner variability, consistent with previous multi-site studies (Magnotta et al., 2012; Palacios et al., 2017), suggesting that it is unlikely that the learning-dependent differences we observed between groups (training versus no-training control group) could be due to differences in data quality. Further, an active training control (i.e., training participants with the same stimuli but on a different task) would be appropriate for testing specificity to the training task. However, selecting the appropriate control task is confounded by the fact that statistical learning has been shown to occur by mere exposure to the stimuli (i.e., without performing a task) and generalize across similar tasks (Shanks, 2004; Perruchet and Pacton, 2006; Turk-Browne and Scholl, 2009; Frost et al., 2015). Further work is needed to translate these basic research findings to effective training programs: future studies employing multi-arm interventions and comparing across groups trained on different tasks are needed to determine which task provides the most effective training, whether brain connectivity changes related to statistical learning are long-lasting, generalize to novel (i.e., untrained) settings and relate to real-life changes in cognitive abilities.

In sum, here we investigated learning-dependent plasticity in brain pathways that mediate statistical learning. Our findings provide evidence that WM connectivity changes with learning to support our ability to extract behaviorally-relevant statistics. This learning-dependent plasticity relates to individual decision strategies, implicating distinct cortico-striatal circuits in learning predictive statistics. Interestingly, these pathways have been previously implicated in reward-based learning (Cohen et al., 2008, 2009; de Wit et al., 2012; Piray et al., 2016), artificial grammar (Flöel et al., 2009), and language learning (Schlegel et al., 2012; Hofstetter et al., 2016). Considering findings across studies, it is possible that common WM pathways subserve learning of temporal structures with feedback or by mere exposure, suggesting potential common brain mechanisms for supervised versus unsupervised learning that may support a range of functions from learning simple temporal contingencies to extracting complex linguistic structures.

## Synthesis

Reviewing Editor: Satu Palva, University of Helsinki Decisions are customarily a result of the Reviewing Editor and the peer reviewers coming together and discussing their recommendations until a consensus is reached. When revisions are invited, a fact-based synthesis statement explaining their decision and outlining what is needed to prepare a revision will be listed below.

I have evaluated your resubmitted manuscript, and your rebuttal to the critique raised by the reviewers together with the editorial board members as well as with experts in the field. We feel that you have adequately addressed the criticism raised by the reviewers and we believe that your results on white matter connectivity reflect true training effects.
